# Effects of Virgin Olive Oils Differing in Their Bioactive Compound Contents on Biomarkers of Oxidative Stress and Inflammation in Healthy Adults: A Randomized Double-Blind Controlled Trial

**DOI:** 10.3390/nu11030561

**Published:** 2019-03-06

**Authors:** Estefania Sanchez-Rodriguez, Sara Biel-Glesson, Jose R. Fernandez-Navarro, Miguel A. Calleja, Juan A. Espejo-Calvo, Blas Gil-Extremera, Rafael de la Torre, Montserrat Fito, Maria-Isabel Covas, Pedro Vilchez, Juan de Dios Alche, Emilio Martinez de Victoria, Angel Gil, Maria D. Mesa

**Affiliations:** 1 Department of Biochemistry and Molecular Biology II, Institute of Nutrition and Food Technology “José Mataix”, Biomedical Research Center, University of Granada, Parque Tecnológico de la Salud, Avenida del Conocimiento s/n, Armilla, 18016 Granada, Spain; estefaniasr@outlook.com (E.S.-R.); agil@ugr.es (A.G.); 2 Fundación Pública Andaluza para la Investigación Biosanitaria de Andalucía Oriental “Alejandro Otero” (FIBAO), Avenida de Madrid 15, 18012 Granada, Spain; sbiel@fibao.es (S.B.-G.); estrategia@innofood.es (J.R.F.-N.); mangel.calleja.sspa@juntadeandalucia.es (M.A.C.); 3 Instituto para la Calidad y Seguridad Alimentaria (ICSA), Avenida de la Hispanidad 17, 18320 Santa Fe, Granada, Spain; jaespejo@hotmail.com; 4 Department of Medicine, University of Granada, Avenida de la Investigación 11, Armilla, 18016 Granada, Spain; Blasgil@ugr.es; 5 Integrative Pharmacology and Systems Neuroscience Research Group, IMIM (Hospital del Mar Research Institute), Universitat Pompeu Fabra (CEXS-UPF), Dr. Aiguader 88, 08003 Barcelona, Spain; rtorre@imim.es; 6 Spanish Biomedical Research Networking Centre, Physiopathology of Obesity and Nutrition (CIBEROBN), Instituto de Salud Carlos III, Monforte de Lemos 3-5, 28029 Madrid, Spain; mfito@imim.es (M.F.); Maria.nuproas@gmail.com (M.-I.C.); 7 Cardiovascular Risk and Nutrition Research Group, Hospital del Mar Medical Research Institute (IMIM), Barcelona, Spain, Dr. Aiguader 88, 08003 Barcelona, Spain; 8 NUPROAS Handelsbolag, Nackã, Sweden, NUPROAS HB, Apartado de Correos 93, 17242 Girona, Spain; 9 Laboratorio CEM Europa S.L., Polígono Industrial “Cañada de la Fuente”, Carretera Fuensanta, s/n, 23600 Martos, Jaén, Spain; pvilchez@cmeuropa.com; 10 Department of Biochemistry, Cell and Molecular Biology of Plants, Estación Experimental del Zaidín (CSIC), Profesor Albareda 1, 18008 Granada, Spain; Juandedios.alche@eez.csic.es; 11 Department of Physiology, Institute of Nutrition and Food Technology “José Mataix”, Biomedical Research Center, University of Granada, Parque Tecnológico de la Salud, Avenida del Conocimiento s/n, Armilla, 18100 Granada, Spain; emiliom@ugr.es; 12 Instituto de Investigación Biosanitaria de Granada (ibs.GRANADA). Complejo Hospitalario Universitario de Granada, 18014 Granada, Spain

**Keywords:** olive oil, virgin olive oil, triterpenes, phenolic compounds, cardiovascular diseases, maslinic acid, oleanolic acid, oxidative stress, 8-hidroxy-2′-deoxyguanosine, TNF-alpha, interleukin-8

## Abstract

A regular consumption of virgin olive oil (VOO) is associated with a reduced risk of cardiovascular disease. We aimed to assess whether the raw intake of an optimized VOO (OVOO, 490 ppm of phenolic compounds and 86 ppm of triterpenes), and a functional olive oil (FOO, 487 ppm of phenolic compounds and enriched with 389 ppm of triterpenes) supplementation (30 mL per day) during three weeks would provide additional health benefits to those produced by a standard VOO (124 ppm of phenolic compounds and 86 ppm of triterpenes) on oxidative and inflammatory biomarkers. Fifty-one healthy adults participated in a randomized, crossover, and controlled study. Urinary 8-hidroxy-2′-deoxyguanosine, plasma interleukin-8 (IL-8), and tumor necrosis factor α (TNF- α) concentrations were lower after the intervention with the FOO than after the OVOO (*p* = 0.033, *p* = 0.011 and *p* = 0.020, respectively). In addition, IL-8 was lower after the intervention with FOO than after VOO intervention (*p* = 0.002). This study provides a first level of evidence on the in vivo health benefits of olive oil triterpenes (oleanolic and maslinic acids) in healthy humans, decreasing DNA oxidation and plasma inflammatory biomarkers. The trial was registered in ClinicalTrials.gov ID: NCT02520739.

## 1. Introduction

The excessive formation of reactive oxygen species (ROS) causes extensive damage to cellular macromolecules, such as polyunsaturated lipids, proteins, and DNA. Oxidative stress processes are associated with cardiovascular diseases (CVD) and other noncommunicable chronic diseases, such as obesity, type-II diabetes, and cancer [1]. CVD represent the main cause of disability and death in industrialized countries. Atherosclerosis, the major physiopathological cause of CVD, is a multifactorial disease involving inflammatory, oxidative, proliferative, and necrotic processes into the arterial walls. This chronic inflammatory condition activates immune competent cells in lesions producing mainly proinflammatory cytokines [2]. The Mediterranean diet is recognized as a healthy food pattern [3]. Olive oil, the main source of fat of the Mediterranean diet, is shown to have antioxidant properties [4] and is associated with a lower incidence of chronic inflammatory diseases such as CVD [4,5,6] due to the antioxidant properties of its minor compounds [7].

Besides the high content of monounsaturated oleic acid, other minor bioactive components are described in virgin olive oil (VOO) [8]. Components from the polar fraction include mainly phenolic compounds, while triterpenic acids stand out in the unsaponifiable one, among others. The presence of these molecules depends on the olive ripeness, the cultivar type, the climate of the area of cultivation, and the type of oil extraction processes [9]. Oleuropein and hydroxytyrosol are the most important active phenols present in VOO [10]. In 2011, the European Food Safety Authority (EFSA) released a health claim concerning the benefits of the daily intake of VOOs, containing at least 5 mg of hydroxytyrosol and related compounds (i.e., oleuropein and tyrosol) per 20 g of VOO, decreasing low density lipoprotein cholesterol (LDLc) oxidation [11]. This implies that only VOOs with a high phenolic content can claim the benefits. Therefore, the necessity of optimize the VOO processing in order to obtain bioactive compound contents is one of the current goals in terms of increasing its nutritional value. Among minor VOO compounds, triterpenic acids also have potential health benefits against CVD and their complications [12,13,14]. The highest content of triterpenic acids is found in the seeds and the skin of the olives, which are usually used for pomace olive oil production. Among olive oil triterpenic acids, oleanolic and maslinic show anticancer [15], anti-inflammatory [16,17], antioxidant [18] and cardioprotective [19] activities in cellular and animal models [20,21]. However, to the best of our knowledge, the NUTRAOLEUM study is the first human study focused on providing evidence of the health benefits of olive oil triterpenes. Due to this, within the frame of this study, we aimed to evaluate the effect of VOOs enriched with bioactive compounds, such as phenolic compounds and triterpenes, on the oxidative and inflammatory biomarkers in healthy adults.

## 2. Materials and Methods

### 2.1. Subjects

The study design, sample size, and the characteristics of the healthy adults were described in detail previously [22]. The flow chart, aspects of their diet during interventions, compliance of the study, and the nutritional composition of the experimental oils administered were also previously published [19]. In brief, 58 intention-to-treat subjects aged from 20 to 50 years were eligible from the general population of Granada, but only 53 subjects (27 men and 26 women) accepted to participate. They were then enrolled in the study from February 2014 to July 2014 and were assigned into groups. Two subjects did not complete the study. After the first intervention, one subject refused to continue for personal reasons, and the other did not follow the protocol correctly. Fifty-one of them completed the study. The inclusion criteria were as follows: People in good health on the basis of a physical examination and basic biochemical and hematological analyses, and willingness to provide written informed consent. The exclusion criteria were as follows: Smoking, intake of antioxidant supplements, aspirin, or any other drug with established antioxidant properties, hyperlipidemia, obesity (body mass index (BMI) > 30 kg/m^2^), diabetes, hypertension, celiac disease, or other intestinal disease, any condition limiting mobility, life-threatening diseases, or any other disease or condition that would impair compliance. The local institutional review board (Ethics Committee Research Center of Granada), approved the protocol (13/11 C38). The trial was registered in ClinicalTrials.gov ID: NCT02520739. All subjects provided written informed consent according to the principles of the Declaration of Helsinki.

### 2.2. Study Design

The NUTRAOLEUM study was a randomized, crossover, controlled, and double-blind study over three weeks of intervention with three different oils and two weeks of washout between the three interventions. The trial was conducted in Virgen de las Nieves and San Cecilio General Hospitals of Granada, Spain. We randomly assigned subjects, paired by gender and age, consecutively to one of three sequences of olive oil administration, using random numbers generated by a computerized generator using the block-randomization method of a software program for sequence generation by an independent statistician. The randomization list was concealed in a lightproof sealed envelope. The sealed envelope was kept by the independent statistician of the study. Participants, investigators, and outcome assessors were blind to the treatment allocation throughout the course of the study.

Each group of subjects (*n* = 18) received a daily dose of 30 mL (28 g) of one olive oil with different amounts of bioactive compounds: (1) An optimized VOO (OVOO) high in phenolic compounds (490 ppm of phenolic compounds, and 86 ppm of triterpenes) produced from Picual olives; (2) A functional olive oil (FOO), that was the same OVOO high in phenolic compounds (487 ppm) and enriched with triterpenes (389 ppm) from olive exocarp, providing 4.7 mg of oleanolic acid and 6 mg of maslinic acid per day (after 30 mL of olive oil); and (3) A VOO obtained from the OVOO after washing to eliminate the majority of phenolic compounds (124 ppm of phenolic compounds, and 86 ppm of triterpenes) (Table 1). San Francisco de Asís Cooperative provided the olive oils used in the present study (Andalucía, Spain). In addition, subjects were instructed to consume the corresponding oil in replacement of other raw fats during the period of intervention. In the two-week washout period prior to interventions, subjects were requested to avoid olives and olive oil consumption and were provided with sunflower oil for cooking and raw purposes. Subjects were requested to avoid the intake of foods containing high amounts of antioxidants. A nutritionist personally advised subjects for replacing all types of regularly consumed raw fats using only the assigned oil.

### 2.3. Evaluation of Dietary Intake

Subjects completed a three-day dietary record at baseline and during each intervention period [23]. Energy consumption and dietary intakes of macro and micronutrients data were processed by using a CSG software (General ASDE) and the Spanish Food Composition Database [19,24].

### 2.4. Blood Sample Collection

Fasting venous blood samples were collected at the beginning of the study (baseline) and before (pre-intervention, after the washout period) and at the end (post-intervention) of each olive oil intervention period using EDTA-coated tubes. Blood samples were centrifuged (4 °C, 10 min at 1750× *g*), and aliquots of plasma were immediately frozen and stored at −80 °C until analysis. Twenty-four hours urine was also collected, homogenized, frozen, and stored at −80 °C until analysis.

### 2.5. Primary Outcomes

#### 2.5.1. Measurement of Urinary 15-F2t-isoprostane and 8-hidroxy-2′-deoxyguanosine Concentrations

Urinary 15-F2t-isoprostane was determined using commercial competitive ELISA kit (EA85 Oxford Biomedical Research, Michigan, USA) (intra-assay coefficient of variation (CV): 14.13%). 8-Hidroxy-2′-deoxyguanosine (8-OHdG) was also determined by using commercial competitive ELISA kit (KOG-200S/E JaICA, Fukuroi, Japan) (CV: 5.73%). The concentrations were normalized by urinary creatinine and expressed in ng/mg of creatinine. Creatinine concentration in urine samples was determined with a colorimetric kit (Ref. 1001115, Spinreact, Spain) (CV: 2.89%).

#### 2.5.2. Measurement of Plasma Inflammation Biomarkers

Plasma interleukin-8 (IL-8) and tumor necrosis factor-α (TNF-α) concentrations were determined by using a BIO-PLEX PRO Human Chemokine Kit (Bio-Rad Laboratories S.A., Madrid, Spain), according to the manufacturer’s instructions (Cat. YZ0-0008J28BE66), in conjunction with a Luminex^®^ 200 system with the XMap technology (Luminex Corporation, Austin, TX, USA) (CV of IL-8 and TNF-α: 10.93% and 10.71%, respectively).

### 2.6. Secondary Outcomes

#### 2.6.1. Measurement of Plasma Biomarkers of the Non-Enzymatic Antioxidant Defense System

Plasma concentrations of retinol, tocopherols, carotenes, coenzyme Q-9 (CoQ_9_), and coenzyme Q-10 (CoQ_10_) were determined after extraction with 1-propanol by ultra-high-pressure liquid chromatography coupled to mass spectrometry (UHPLC-MS), using methanol 0.1% and isocratic formic as solvent with a flow of 0.5 mL/min in a ACQUITY UPLCr BEH C18 50 mm column (internal diameter 2.1 mm and particle size 1.7 μm) [25]. Concentrations are given in µg/L for CoQ_9_ and in mg/L for the rest of analytes.

#### 2.6.2. Measurement of Fatty Acids in Plasma

The fatty acids profile was quantified in plasma samples after methylation by gas-liquid chromatography coupled with mass spectrometry [26] using a 30 m long capillary column (ZB-FAME, 0.25 mm internal diameter and 0.25 µm film thickness) (Supelco, Bellefonte, CA, USA). Fatty acid methyl esters from plasma lipids were obtained as previously reported by Lepage and Roy (1986) [27]. Briefly, the hexane extracts of total plasma and lipid fractions were dissolved into 2 mL methanol:benzene (4:1 *v*/*v*). Methylation was carried out at 100 °C for 1 h by adding 200 mL acetyl chloride. After cooling, 5 mL of 0.43 M K_2_CO_3_ was added to stop the reaction and neutralize the mixture. Tubes were then shaken and centrifuged and the benzene upper phase was dried under N_2_ and resuspended with 100 µL hexane.

### 2.7. Sample Size and Power Analysis

Accepting an alpha risk of 0.05 and a beta risk of 0.20 in a two-sided test, at least 40 subjects in the sustained consumption study are necessary to recognize as statistically significant a difference greater than or equal to 10 units in the oxidized LDL. A dropout rate of 15% was anticipated. This sample size also permits to show, as statistically significant with a power of 80%, a difference of 10 lag-time units among treatments in the clinical trial. Dropouts before the first intervention period were replaced in order to avoid dropouts greater than 15%. We tried to enlarge the sample size to 54 in order to have more statistical power for our results.

### 2.8. Statistical Analyses 

Baseline data are presented as the mean values ± standard error of the mean (SEMs) unless otherwise indicated. The normality of variables was assessed using Q-Q graphs. The homogeneity of the variances was estimated by using Levene’s test. One-factor ANOVA or Kruskal-Wallis tests were used for continuous variables (depending on whether the normality assumption was met) to determine differences among the three olive oil interventions and in terms of baseline characteristics. 

Biochemical parameters are presented as adjusted mean values ± SEMs and were analyzed using a linear mixed-effects model (LMM). Missing data were imputed using appropriate methods. The outliers for each intervention were removed if kurtosis >1 and asymmetry >1 in the distribution of the responses. Variables that did not follow a normal distribution confirmed by skewed distribution (Q-Q graphs) were log-transformed (15-F2t-isoprostane, 8-OHdG, CoQ_9_, CoQ_10_, carotenes, linolenic acid and eicosapentaenoic acid). An LMM adjusted by age, gender, pre-intervention, and period as fixed effects and for subjects and hospital as random effects [28] was used to compare variables before and after each intervention (pre- vs. post-interventions, intra-treatment effect) and to compare interventions between the groups after the three-week intervention (intertreatment effect). Multiple comparisons post hoc analysis are given using the Sidak test. The carryover effects were assessed as the interaction between period and intervention [28] to check the effectiveness of the washout periods. In addition, an interaction term was checked for differences on the effect part intervention by gender. The same model was also used to compare changes of the variables (post-intervention minus pre-intervention) without adjusting for pre-intervention. Correlations between variables were estimated by the Pearson’s correlation coefficient when the assumptions of normality were met and by the Spearman’s correlation coefficient when the assumptions of normality were not met. We performed all analysis on an intention-to-treat basis. A *p* < 0.05 value was considered significant. Statistical Package for the Social Sciences version 20 software was used to perform the statistical analysis (SPSS Inc, Chicago, IL, USA). 

## 3. Results

### 3.1. Baseline Characteristics

Clinical and biochemical characteristics of subjects at the beginning of the study according to the type of olive oil administration sequence as well as energy, macronutrient, and main antioxidants intakes were previously described [19] and are described in supplementary Table S1. In brief sequences were: Sequence 1: OVOO, VOO, and FOO; Sequence 2: VOO, FOO, and OVOO; Sequence 3: FOO, OVOO, and VOO. No significant differences were observed among the three groups of intervention at baseline of the study. Table 2 shows the profile of plasma fatty acids, urinary oxidative stress biomarkers, plasma biomarkers of the non-enzymatic antioxidant defense system, and inflammation at baseline according to the sequence of olive oil administration (grouping criteria). Differences between baseline characteristics of volunteers from Sequences 1 and 2 were observed for CoQ_9_ and CoQ_10_ (*p* = 0.002 and *p* = 0.015, respectively). Differences between the subjects from Sequences 1 and 3 were observed for carotenes (*p* = 0.005). Differences between the subjects from Sequences 2 and 3 were observed for 15-F2t-isoprostane (*p* = 0.025) and carotenes (*p* = 0.022). In addition, differences were observed for IL-8 between subjects from the Sequences 1 vs. 2 and 3 (*p* = 0.005, *p* = 0.028, respectively).

### 3.2. Plasma Fatty Acids Profile

Table 3 shows the profile of the main plasma fatty acids before and after the three interventions. The percentage of plasma unsaturated oleic acid (C18:1 n-9) increased after three weeks of intervention with the three olive oils (all *p* < 0.001), while linoleic acid (C18:2 n-6) significantly decreased after the three-week intervention with the VOO and with the OVOO (all *p* < 0.001), and had a trend to decrease after the FOO (*p* = 0.054). Percentages of stearic (C18:0), palmitic (C16:0), linolenic (C18:3 n-3), arachidonic (C20:4 n-3), eicosapentaenoic (EPA, C20:5 n-3), and docosahexaenoic (DHA, C22:6 n-3) acids did not change after the three interventions. No significant intertreatments differences were found. 

### 3.3. Urinary Oxidative Stress Biomarkers

**Figure 1:**
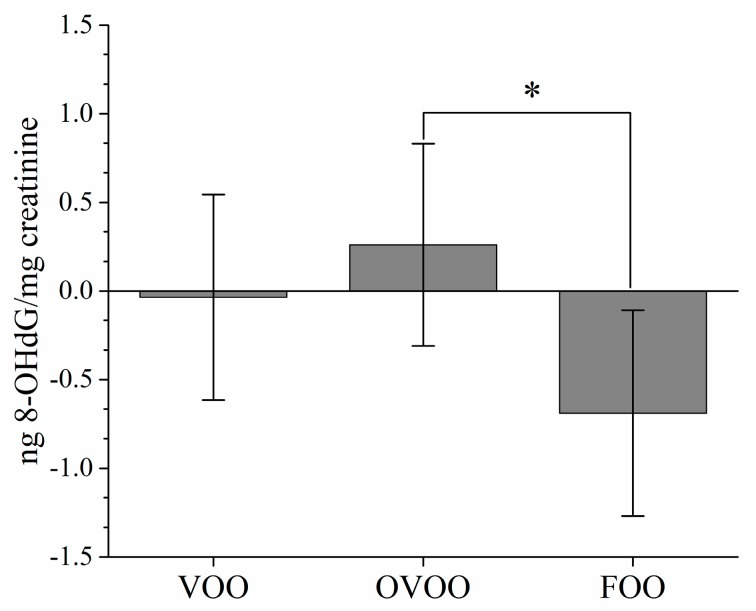
Post-pre-intervention changes in urinary 8-OHdG in a randomized control trial evaluating the effects of virgin olive oils differing in their bioactive compound contents on oxidative stress and inflammation. * Indicates significant differences (*p* < 0.05). FOO, functional olive oil; 8-OHdG, 8-hidroxy-2′-deoxyguanosine; OVOO, optimized virgin olive oil; VOO, virgin olive oil.

Table 4 shows urinary oxidative stress biomarkers concentrations before and after the three interventions. When comparing pre- vs. post-intervention (intra-treatment effect), urinary 8-OHdG levels did not change after the three-week intervention periods (Table 4). However, when analyzing the intertreatment effects, urinary 8-OHdG levels tended to be lower after the intervention with the FOO than after the VOO intervention (*p* = 0.068; Table 4). In addition, the levels of urinary 8-OHdG were lower in males than in females (*p* < 0.001) in all interventions (data not shown), although no interaction was observed between gender and intervention. Urinary 8-OHdG levels change (i.e., decrease post-intervention minus pre-intervention) induced by the FOO was higher than that induced after the OVOO intervention (*p* = 0.033, Figure 1). 

Urinary 15-F2t-isoprostane maintained unchanged after the VOO, OVOO and FOO interventions (*p* = 0.053, *p* = 0.070 and *p* = 0.179, respectively; Table 4). However, a negative correlation was observed between plasma oleic acid and urinary 15-F2t-isoprostane (*r* = −0.132; *p* = 0.015). 

### 3.4. Non-Enzymatic Antioxidant Defense System

Table 4 shows some antioxidant compounds levels in plasma samples. Retinol, tocopherols, CoQ_9_, CoQ_10_, and carotenes concentrations did not show changes after the three interventions. When analyzing the intertreatment effects, no differences were found between interventions. Males had higher levels of plasma retinol (0.22 ± 0.01 mg/dL) than females (0.20 ± 0.01 mg/dL) for all interventions (*p* = 0.014). 

### 3.5. Plasma Inflammation Biomarkers

Table 4 show plasma inflammatory biomarkers levels before and after the three interventions in healthy adults. Plasma inflammatory biomarkers did not change after the three interventions, but when comparing inter-treatment effects, IL-8 levels were lower after the intervention with FOO than after VOO (*p* = 0.002) and OVOO (*p* = 0.011). Plasma TNF-α levels lowered only after the intervention with the FOO than after the OVOO (*p* = 0.020). The effect was mainly observed in females but the interaction between gender and interventions was not significant.

## 4. Discussion

The present study provides a first level of evidence on the in vivo cardiovascular benefits of olive oil triterpenes (oleanolic and maslinic acids) in healthy humans, decreasing plasma inflammatory biomarkers and DNA oxidation after three weeks of supplementation. Olive oil is a good food matrix to be a vehicle for triterpenes, as they naturally contain these compounds but in low amounts. Experimental studies on olive oil triterpenic acids, from the unsaponifiable fraction, showed a number of benefits against CVD and their complications [12,13,14,29,30]. The fatty acid profiles after the three interventions confirm the adherence of the subjects to the interventions since an increase of plasma oleic acid was observed after each of them, reflecting the lipid composition of the diet [31]. The present study shows that a daily supplementation during three weeks with 30 mL of a FOO providing 13.4 mg/d of phenolic compounds (487 ppm) and 4.7 mg/d of oleanolic acid and 6 mg/d of maslinic acid (171 and 218 ppm, respectively) may protect DNA against oxidative stress in vivo, by decreasing urinary 8-OHdG concentrations compared with the same olive oil but without triterpenes. 8-OHdG is an oxidative stress biomarker produced by oxidation of the nucleoside deoxyguanosine and is subsequently excreted directly into the urine. It was described as a sensitive biomarker of oxidative DNA damage [32]. A recent meta-analysis reported that 8-OHdG levels are higher in patients with CVD than in healthy controls [33]. In this regard, on one hand, in vitro [34,35] and animal [36] studies reported that olive oil phenolic compounds protect DNA from oxidation. On the other hand, in vitro studies reported that pentacyclic triterpenes are also able to protect against oxidative DNA damage [37]. The 8-OHdG results of the present study is in agreement with observations made in the EUROLIVE study, in which supplementation with olive oil protects DNA against oxidation after three weeks of supplementation in healthy males [38]. This effect was independent of the phenolic content and related to the monounsaturated fatty acid content of the olive oil [38]. In addition, a protection of DNA oxidation in hyperlipidemic subjects was observed after the intake of a VOO complemented with thyme phenolic compounds [39], and after eight weeks of supplementation with 50 g/d of an olive oil with 592 ppm of phenols in postmenopausal women [40]. Furthermore, 11.13 mg of olive’s phenolic compounds have demonstrated postprandial protection against in vivo DNA oxidation in healthy males after four days of supplementation [41]. All these positive data differ from the null effect of our VOO and OVOO, probably due to the lower amount of phenols contained in the oils used in the present study. In addition, physiological and environmental situations of volunteers may also influence the results. Therefore, all these facts may indicate that a longer intervention with the OVOO may have more impact on healthy adults and also on different pathological situations. More studies are needed in order to validate the positive effect of triterpenic acids-rich olive oils on DNA oxidation. 

F2-isoprostanes are generated in vivo by free radical-induced peroxidation of arachidonic acid [42]. One of the major F2-isoprostanes, 15-F2t-isoprostane increase in several situations associated with oxidative stress, including atherosclerosis, diabetes, obesity, cigarette smoking, neurodegenerative diseases, and asthma [43]. The present study shows that there was no effect on urinary 15-F2t-isoprostane levels after supplementation with VOOs enriched in phenolic and triterpenic compounds in healthy humans. The effect of olive oil phenolic compounds is not fully elucidated. In agreement with our results, a null effect on urinary isoprostanes levels of olive oil phenolic compounds were reported in dyslipidemic subjects [44]. Also, a previous randomized crossover study with a larger sample size reported that lipid peroxidation decreased in a linear manner with the phenolic content of the olive oil, particularly in markers directly associated with LDL oxidation, but not F2-isoprostanes, in healthy subjects [45]. In vitro [46] and in vivo [9,47] experimental studies demonstrated an antioxidant effect of triterpenic acids. Particularly, oleanolic acid showed a substantial protection against in vitro lipid peroxidation in isolated rat liver microsomes [48], as well as in a cerebral ischemic animal model [49]. It was proposed that oleanolic acid is not only a free radical-scavenger acting through direct chemical reactions, but also a biological molecule which may enhance the antioxidant defense [49]. In addition, maslinic acid is a potent inhibitor of oxidative stress and cytokine production in stimulated murine macrophages in vitro [16]. In fact, the negative correlation found between oleic and urinary 15-F2t-isoprostane may suggest that a longer intervention would be able to exert a positive effect on reducing urinary 15-F2t-isoprostane concentrations. This is in agreement with other published preclinical [50] and clinical [51] studies. 

When analyzing plasma non-enzymatic antioxidant defense system, at the beginning of the study, the concentrations of CoQ_9_, CoQ_10_, and carotenes in plasma were different between the three sequences of olive oil consumption, while no further changes were observed after the all interventions. According to the nutritional data registered during the study, our volunteers did not modify the diet. Therefore, a change in plasma antioxidant molecules was not expected. Indeed, the absence of these changes suggests that the improved oxidative status observed after the three olive oil interventions could mainly depend on other macro- or micro- components present in the olive oils. 

The present study shows that daily supplementation during three weeks with 30 mL of a FOO rich in phenolic compounds and enriched with olive triterpenic acids improves plasma inflammatory biomarkers, by decreasing plasma IL-8 and TNF-α concentrations, compared with the oils with less triterpenic acids. This is the first time than an in vivo effect of triterpenic acids from olive oil is described in humans. The OVOO improved the IL-8 concentration, but did not modify the TNF-α. The Mediterranean diet is associated with a lower incidence of chronic inflammatory diseases [6]. A recent review of human studies indicated that olive oil consumption reduces TNF-α and IL-6 levels when associated with Mediterranean diet and lifestyle [52]. A systematic review concluded that olive oil might exert beneficial effects on inflammatory biomarkers, such as C reactive protein and IL-6, after at least four weeks of intervention in individuals adhering to the Mediterranean diet [5]. In addition, long-term adherence to this Mediterranean diet decreased plasma concentrations of IL-6 and IL-8 related to different steps of atheroma plaque development in elderly subjects at high cardiovascular risk [53]. The major anti-inflammatory components in olives are tyrosol, hydroxytyrosol, oleuropein, ligstroside, verbascoside, and their derivatives [54]. In particular, hydroxytyrosol is considered the major anti-inflammatory compound in aqueous olive extracts [55]. In agreement with our results, human studies observed a decrease of IL-8 concentrations after the intake of 136.2 mg of oleuropein and 6.4 mg of hydroxytyrosol per day during an intervention of six weeks in pre-hypertension males [56]. In addition, in vivo and in vitro studies suggest that pentacyclic triterpenes exhibit anti-inflammatory effects [57,58]. 

We chose three weeks of intervention based on a previous study EUROLIVE, which reported the beneficial effects on HDLc and oxidized LDL plasma in a dose-dependent manner of the phenolic content in healthy volunteers from the North of Spain [38]. One of the limitations of the present study is that young and healthy subjects were recruited, and, in addition, our subjects lived in the South of Spain, where Mediterranean diet and VOO are highly consumed. Therefore, a three-week intervention may not be long enough to cause significant changes. Further long-term studies, particularly in patients with CVD and related comorbidities, are required to find conclusions related to the bioactive compounds presents in olive oils, and to explore the mechanisms involved in these effects of specific components of VOO on oxidative stress.

## 5. Conclusions

The presence of oleanolic and maslinic acids from olive seeds in VOO confers protection against inflammation. This effect is observed after supplementation with 30 mL/d of a FOO containing 4.7 mg of oleanolic acid and 6 mg of maslinic acid during three weeks in healthy adults. Additionally, FOO shows antioxidant activity against DNA oxidation compared to the same olive oil but without triterpenes.

## Figures and Tables

**Table 1:** Characteristics of the olive oil administered.

	VOO	OVOO	FOO
Fatty acid profile (%)			
C18:0	2.3	2.2	2.1
C18:1n9	78.9	78.2	78.4
C18:2n6	6.6	6.8	6.9
C18:3n3	0.6	0.7	0.7
C20:0	0.4	0.4	0.4
C20:1	0.3	0.4	0.4
C22:0	0.1	0.1	0.1
C24:0	<0.1	<0.1	<0.1
Total phenolic compounds (ppm)	124	490	487
Hydroxytyrosol and derivates	105	424.0	423.0
Ty-EA	59	156	156
Hy-EA	20	127	124
Ty-EDA	19	81	78
Hy-EDA	3	40	43
Tyrosol	1	10	9
Hydroxytyrosol	1	3	5
Lignanes	18.2	61.3	59.2
Flavonoids	0.7	3.4	3.2
Simple phenols	0.0	0.9	0.9
Total triterpenes (mg/kg)	86.5	86.3	388.8
Maslinic acid	47.3	47.3	217.7
Oleanolic acid	39.2	39.1	171.1
Ursolic acid	<10	<10	<10
α-tocopherol (ppm)	174	183	176
Squalene (mg/100 g)	529.2	536.2	545.5
Total pigments (ppm)	15.73	17.59	16.78
Total carotenoid pigments (ppm)	7.08	6.79	6.97
Total sterols (ppm)	1437	1396	1460

Hy-EA, oleuropein aglycon; Hy-EDA, dialdehydic form of decarboxymethyl oleuropein aglycon; Ty-EA, ligstroside aglycon; Ty-EDA, dialdehydic form of decarboxymethyl ligstroside aglycon; FOO, functional olive oil; OVOO, optimized virgin olive oil; VOO, virgin olive oil.

**Table 2:** Main plasma fatty acids, urinary oxidative stress biomarkers, plasma biomarkers of the non-enzymatic antioxidant defense system, and inflammation at baseline in a randomized control trial evaluating the effects of virgin olive oils differing in their bioactive compound contents on oxidative stress and inflammation.

	Sequence 1	Sequence 2	Sequence 3
**Main Plasma Fatty Acids (%)**			
Palmitic acid	21.0 ± 0.5	21.3 ± 0.7	21.5 ± 0.6
Stearic acid	6.2 ± 0.2	5.7 ± 0.1	5.7 ± 0.2
Oleic acid	20.4 ± 1.1	19.0 ± 1.0	18.2 ± 0.7
Linoleic acid	40.4 ± 1.1	40.9 ± 1.4	41.4 ± 1.0
Linolenic acid	1.4 ± 0.3	2.5 ± 0.4	2.1 ± 0.3
AA	6.1 ± 0.31	5.7 ± 0.3	5.6 ± 0.2
EPA	0.20 ± 0.02	0.14 ± 0.01	0.20 ± 0.02
DHA	0.90 ± 0.06	0.80 ± 0.07	0.90 ± 0.07
**Urine oxidative stress biomarkers**			
8-OHdG, ng/mg creatinine	7.4 ± 0.4	7.6 ± 0.7	8.7 ± 0.7
15-F2t-isoprostane, ng/mg creatinine	2.7 ± 0.3 ^ab^	2.3 ± 0.3 ^a^	3.5 ± 0.3 ^b^
**Plasma biomarkers of the non-enzymatic antioxidant defense system**			
Retinol, mg/L	0.21 ± 0.01	0.18 ± 0.01	0.21 ± 0.01
Tocopherols, mg/L	5.8 ± 0.3	5.2 ± 0.2	5.3 ± 0.2
CoQ_9_, µg/L	96.0 ± 7.0 ^a^	62.0 ± 5.0 ^b^	81.0 ± 7.0 ^a,b^
CoQ_10_, mg/L	1.9 ± 0.1 ^a^	1.4 ± 0.1 ^b^	1.6 ± 0.1 ^a,b^
Carotenes, mg/L	0.25 ± 0.03 ^a^	0.23 ± 0.03 ^a^	0.11 ± 0.03 ^b^
IL-8, pg/mL	2.1 ± 0.2 ^a^	1.3 ± 0.1 ^b^	1.5 ± 0.2 ^b^
TNF-α, pg/mL	2.1 ± 0.2	1.9 ± 0.2	1.9 ± 0.2

Values are expressed as the means ± SEMs. ANOVA was used to compare results between groups. Different letters (^a,b^) indicated significant differences. Sequence 1: OVOO, VOO and FOO olive oil, *n* = 20; Sequence 2: VOO, FOO and OVOO olive oil, *n* = 19; Sequence 3: FOO, OVOO and VOO olive oil, *n* = 19. 8-OHdG, 8-hidroxy-2′-deoxyguanosine; AA, araquidonic acid; CoQ_9_, coenzyme Q-9; CoQ_10_, coenzyme Q-10; DHA, docosahexaenoic acid; EPA, eicosapentaenoic acid; FOO, functional olive oil; IL-8, interleukin-8; OVOO, optimized virgin olive oil; SEM, standard error of the mean; TNF-α, tumor necrosis factor-α; VOO, virgin olive oil.

**Table 3:** Main plasma fatty acids percentages before and after the three interventions in a randomized control trial evaluating the effects of virgin olive oils differing in their bioactive compound contents on oxidative stress and inflammation.

	VOO		OVOO		FOO	
Fatty Acids (%)	Pre-Intervention	Post-Intervention	Pre-Intervention	Post-Intervention	Pre-Intervention	Post-Intervention
Palmitic acid	20.5 ± 1.1	20.3 ± 1.1	20.9 ± 1.1	20.8 ± 1.1	21.7 ± 1.1	20.7 ± 1.1
Stearic acid	5.3 ± 0.4	5.2 ± 0.4	5.3 ± 0.4	5.4 ± 0.4	5.4 ± 0.4	5.1 ± 0.4
Oleic acid	16.0 ± 1.5	22.0 ± 1.5 *	16.8 ± 1.5	21.6 ± 1.5 *	16.5 ± 1.5	20.3 ± 1.5 *
Linoleic acid	47.4 ± 2.5	41.2 ± 2.6 *	46.9 ± 2.5	41.5 ± 2.6 *	46.0 ± 2.5	43.6 ± 2.6
Linolenic acid	0.18 (0.04–0.58)	0.20 (0.07–0.53)	0.17 (0.01–0.79)	0.14 (0.05–0.46)	0.19 (0.08–0.37)	0.24 (0.12–0.56)
AA	6.1 ± 0.4	5.8 ± 0.4	5.6 ± 0.4	5.6 ± 0.4	5.6 ± 0.4	5.7 ± 0.4
EPA	0.17 ± 0.03	0.19 ± 0.03	0.16 ± 0.03	0.17 ± 0.03	0.18 ± 0.03	0.17 ± 0.03
DHA	0.83 ± 0.09	0.87 ± 0.09	0.83 ± 0.09	0.83 ± 0.01	0.87 ± 0.09	0.88 ± 0.01

Values are expressed as adjusted means ±SEMs or as adjusted medians (range) for non-normal variables. Linear mixed-effects model (LMM) was used to compare data after the three interventions (post-interventions) and to compare pre-intervention vs. post-interventions with each oil data. (*) show significant differences (*p* < 0.001) between pre-intervention and post-intervention. AA, arachidonic acid; DHA, docosahexaenoic acid; EPA, eicosapentaenoic acid; FOO, functional olive oil; OVOO, optimized virgin olive oil; VOO, virgin olive oil.

**Table 4:** Urinary oxidative stress biomarkers, plasma biomarkers of the non-enzymatic antioxidant defense system, and inflammatory biomarkers concentrations in a randomized control trial evaluating the effects of virgin olive oils differing in their bioactive compound contents on oxidative stress and inflammation.

	VOO		OVOO		FOO	
	Pre-Intervention	Post-Intervention	Pre-Intervention	Post-Intervention	Pre-Intervention	Post-Intervention
Urine oxidative stress biomarkers						
8-OHdG, ng/mg creatinine	7.8 (5.5–12.0) ^a^	7.3 (5.1–11.3)	7.0 (4.3–10.4) ^b^	7.3 (5.0–11.0)	7.2 (4.8–11.1) ^a,b^	6.7 (4.2–10.3)
15-F2t-isoprostane, ng/mg creatinine	2.3 (1.3–3.7)	2.4 (1.1–4.3)	2.4 (1.2–3.9)	2.4 (0.9–4.3)	2.5 (1.2–3.9)	2.5 (1.0–4.4)
Plasma biomarkers of the non-enzymatic antioxidant defense system						
Retinol, mg/L	0.21 ± 0.01	0.21 ± 0.01	0.21 ± 0.01	0.21 ± 0.01	0.21 ± 0.01	0.21 ± 0.01
Tocopherols, mg/L	5.5 ± 0.2	5.3 ± 0.2	5.5 ± 0.2	5.6 ± 0.2	5.5 ± 0.2	5.5 ± 0.2
CoQ_9_, µg/L	78 (43–141)	74 (34–124)	93 (37–139)	79 (36–129)	85 (41–142)	76 (35–125)
CoQ_10_, mg/L	1.6 ± 0.1	1.6 ± 0.1	1.7 ± 0.1	1.7 ± 0.1	1.7 ± 0.1	1.7 ± 0.1
Carotenes, mg/L	0.17 ± 0.01	0.18 ± 0.01	0.18 ± 0.01	0.18 ± 0.01	0.18 ± 0.01	0.18 ± 0.01
Inflammatory biomarkers						
IL-8, pg/mL	1.7 ± 0.2	1. 8 ± 0.2 ^a^	1.5 ± 0.2	1.7 ± 0.2 ^a^	1.6 ± 0.2	1.4 ± 0.2 ^b^
TNF-α, pg/mL	2.0 ± 0.2	2.0 ± 0.2 ^a,b^	1.9 ± 0.2	2.1 ± 0.2 ^a^	1.9 ± 0.2	1.8 ± 0.2 ^b^

Values are expressed as adjusted means ±SEMs or as adjusted medians (range) for non-normal variables. LMM was used to compare data after the three interventions (post-interventions) and to compare pre-intervention vs. post-interventions with each oil data. Comparing post-interventions data, different superscript letters indicated significant differences (^a,b^). *p* < 0.05 was considered significant. 8-OHdG, 8-hidroxy-2′-deoxyguanosine; CoQ_9_, coenzyme Q-9; CoQ_10_, coenzyme Q-10; FOO, functional olive oil; IL-8, interleukin-8; OVOO, optimized virgin olive oil; TNF-α, tumor necrosis factor-α; VOO, virgin olive oil.

## Supplementary Materials

The following are available online at https://www.mdpi.com/2072-6643/11/3/561/s1, Table S1: Clinical and biochemical characteristics of subjects at baseline according to olive oil administration sequence.
