# Supplementary material for: Effects of Virgin Olive Oils Differing in Their Bioactive Compound Contents on Biomarkers of Oxidative Stress and Inflammation in Healthy Adults: A Randomized Double-Blind Controlled Trial
Source: Nutrients. 2019 Mar 6;11(3):561. doi: 10.3390/nu11030561 (PMC6470869; doi:10.3390/nu11030561)
Supplement: Supplementary file 1 [file nutrients-11-00561-s001.pdf]

Supplementary Table 1. Clinical and biochemical characteristics of subjects at baseline according to olive oil administration sequence.

Characteristics	Sequence 1	Sequence 2	Sequence 3
Age, years	32 ± 2	29 ± 2	28 ± 2
Gender, male n (%)	12 (60)	10 (53)	8 (42)
BMI, kg/m^2	24 ± 1	24 ± 1	24 ± 1
Waist circumference, cm	80 ± 2	78 ± 3	77 ± 2
Males	80 ± 3	82 ± 4	82 ± 2
Females	81 ± 5	73 ± 3	73 ± 2
Glucose, mg/dL	90 ± 2	92 ± 2	87 ± 2
HDLc, mg/dL	58 ± 2	58 ± 3	59 ± 2
Males	55 ± 3	51 ± 3	52 ± 3
Females	64 ± 2	63 ± 5	64 ± 3
LDLc, mg/dL	117 ± 9	107 ± 7	102 ± 5
Total cholesterol, mg/dL	192 ± 10	180 ± 7	175 ± 7
Triglycerides mg/dL	87 ± 7	81 ± 13	67 ± 5
SBP, mmHg	121 ± 2	120 ± 3	118 ± 3
DBP, mmHg	77 ± 2	74 ± 2	71 ± 2
Pulse pressure, mmHg	44 ± 2	46 ± 3	47 ± 2

Values are expressed as means ± SEMs. ANOVA and χ^2 test were used to compare results between groups. Sequence 1: OVOO, VOO and FOO, *n* = 20; Sequence 2: VOO, FOO and OVOO, *n* = 19; Sequence 3: FOO, OVOO and VOO, *n* = 19. BMI, body mass index; DBP, diastolic blood pressure; FOO, functional olive oil; HDLc, high density lipoprotein cholesterol; LDLc, low density lipoprotein cholesterol; SBP, systolic blood pressure; SEM, standard error of the mean; VOO, virgin olive oil.
